# Longitudinal immune trajectories following SARS-CoV-2 vaccination in long-term care facility residents: an unsupervised analysis approach

**DOI:** 10.3389/fragi.2026.1963384

**Published:** 2026-10-19

**Authors:** Annapina Palmieri, Cecilia Damiano, Pasqualina Leone, Caterina Trevisan, Alba Malara, Alberto Zucchelli, Raffaele Antonelli Incalzi, Rosa Prato, Francesca Fortunato, Tatjana Baldovin, Alessandra Coin, Pasquale Minchella, Stefania Giordano, Anna Teresa Palamara, Graziano Onder, Giorgio Fedele

**Affiliations:** 1 Department of Cardiovascular, Endocrine-Metabolic Diseases and Aging, Istituto Superiore di Sanità, Rome, Italy; 2 Department of Infectious Diseases, Istituto Superiore di Sanità, Rome, Italy; 3 Department of Neurobiology, Care Sciences and Society, Karolinska Institutet and Stockholm University, Aging Research Center, Stockholm, Sweden; 4 Department of Medical Sciences, University of Ferrara, Ferrara, Italy; 5 ANASTE Humanitas Foundation, Rome, Italy; 6 Department of Clinical and Experimental Sciences, University of Brescia, Brescia, Italy; 7 Research Unit of Internal Medicine, Department of Medicine and Surgery, Università Campus Bio-15 Medico, Rome, Italy; 8 Department of Medical and Surgical Sciences, Hygiene Unit, Policlinico Foggia Hospital, University of Foggia, Foggia, Italy; 9 Unit of Hygiene and Public Health, Department of Cardiac, Thoracic, Vascular Sciences, and Public, University of Padua, Padua, Italy; 10 Department of Medicine (DIMED), University of Padua, Padua, Italy; 11 Microbiology and Virology Unit, “Pugliese-Ciaccio” Hospital, Catanzaro, Italy; 12 Fondazione Policlinico Universitario “Agostino Gemelli” IRCCS, Rome, Italy; 13 Università Cattolica del Sacro Cuore, Rome, Italy

**Keywords:** K-means clustering, long-term care facilities, principal component analysis, SARS-CoV-2, vaccine

## Abstract

**Background:**

Residents of Long-Term Care Facilities (LTCFs) represent a highly vulnerable population characterized by immunosenescence, multimorbidity, and heterogeneous immune responses to repeated SARS-CoV-2 vaccination and infection. Conventional analyses based on single clinical variables often fail to capture this complexity. We evaluated longitudinal humoral immune responses in institutionalized older adults to identify data-driven antibody profiles and characterize cluster-specific waning kinetics.

**Methods:**

In a prospective multicenter cohort of 388 Italian LTCF residents followed across four time points (T0–T3 over 12 months), longitudinal anti-Spike (anti-S) and anti-Nucleocapsid (anti-N) IgG levels were measured alongside demographic and vaccination data. Principal Component Analysis (PCA) was used for dimensionality reduction, followed by K-means clustering. Optimal cluster number was determined using internal validation metrics (Silhouette, Calinski-Harabasz, and Davies-Bouldin indices) and bootstrap stability analysis. Linear mixed-effects models were applied to describe cluster-specific antibody waning trajectories.

**Results:**

A longitudinal analysis was conducted on 388 LTCF residents. Three antibody trajectory profiles emerged: (i) a Vaccine-Driven profile (n = 130), characterized by moderate anti-S titers and progressive antibody decline; (ii) a Hybrid Immunity profile (n = 125), characterized by evidence of prior infection and remarkably stable antibody concentrations throughout follow-up despite limited uptake of the 2023/2024 booster; and (iii) a High-Peak Hybrid Responder profile (n = 133), exhibiting the highest baseline anti-S titers followed by the steepest rate of antibody waning. Across the cohort, 12 of 388 residents (3.1%) developed documented symptomatic SARS-CoV-2 infection during follow-up, with no COVID-19-related deaths and no significant difference in infection incidence among profiles. Bootstrap stability analysis showed a mean Adjusted Rand Index (ARI) of 0.74 (SD 0.20).

**Conclusion:**

Unsupervised machine learning effectively resolves the complex heterogeneity of SARS-CoV-2 humoral responses in LTCF residents into data-driven trajectory patterns. These findings illustrate the utility of data-driven approach for describing humoral dynamics while distinguishing observational antibody kinetics from direct estimates of clinical protection.

## 1 Introduction

Older adults have suffered a considerable burden of morbidity and mortality throughout the COVID-19 pandemic, with the highest fatality rates observed among individuals aged ≥75 years and residents of Long-Term Care Facilities (LTCFs) (Bonanad et al., 2020; Abidin et al., 2025; Palmieri et al., 2020; Hägg et al., 2020; Sepulveda et al., 2020). Although mRNA vaccines dramatically reduced severe outcomes, humoral immunogenicity in frail older populations is lower and less durable due to immunosenescence and multimorbidity (Tamandjou et al., 2023; Fedele et al., 2022a; Nanishi et al., 2022; Wrona et al., 2024; Franceschi and Campisi, 2014). These immunological changes lead to reduced antibody persistence following vaccination (Crooke et al., 2019; Wagner and Weinberger, 2020; Fedele et al., 2023; Canaday et al., 2022; Fedele et al., 2022b; Dyer et al., 2022). Repeated booster campaigns and varying histories of natural infection have introduced an additional layer of complexity through hybrid immunity (Kometani et al., 2024; Crotty, 2021). Across age groups, hybrid immunity has been associated with higher antibody titers, broader neutralizing capacity against viral variants, and improved protection against severe disease compared with vaccination alone (Hall et al., 2022; Nordströ et al., 2022; Cerqueira-Silva et al., 2022). In older adults, prior SARS-CoV-2 infection has been shown to significantly enhance post-vaccination humoral responses and slow antibody waning (Fedele et al., 2022b; Dyer et al., 2022). Real-world longitudinal studies have provided valuable insights into post-vaccination immunity, yet unraveling the drivers of individual response remains challenging. In complex clinical cohorts, antibody responses exhibit marked heterogeneity, with some individuals mounting robust, sustained responses and others showing rapid waning, that cannot be easily disentangled by evaluating single demographic or clinical variables in isolation. In this context, unsupervised machine learning techniques, such as Principal Component Analysis (PCA) and K-means clustering, provide a practical framework to integrate overlapping exposure histories and longitudinal serological measurements. This data-driven approach allows for the identification of recurrent, data-driven response profiles without relying on predefined clinical assumptions. Additionally, observing these serological trajectories remains conceptually distinct from directly measuring clinical protection against infection. Within this context, early findings from the Italian multicenter GeroCovid Vax study (Abbatecola et al., 2022) provided important real-world evidence on SARS-CoV-2 vaccine responses in LTCFs residents (Fedele et al., 2022a; Fedele et al., 2022b; Virgilio et al., 2022; Trevisan et al., 2023a; Trevisan et al., 2023b; Schiavoni et al., 2023). Despite substantial progress, several critical gaps remain. The long-term kinetics of SARS-CoV-2 humoral immunity after multivaccination in institutionalized older adults remain incompletely characterized, as well as the relative contributions of vaccination, natural infection, and their interaction to antibody persistence and waning in this population. The primary aim of this study was to identify data-driven antibody trajectory profiles and characterize cluster-specific waning kinetics over 12 months in a prospective multicenter cohort of Italian LTCF residents using an unsupervised machine learning approach, and to describe the clinical and immunological characteristics associated with these trajectory profiles.

## 2 Methods

### 2.1 Study design and data collection

This study was conducted within the framework of the COVAC-3 project a prospective, multicenter, longitudinal cohort study conducted in Italy across 13 LTCFs in the regions of Calabria, Friuli-Venezia Giulia, Lazio, Puglia, and Veneto. Residents aged 65 years or older were enrolled between November 2023 and February 2024 and followed for 12 months, with serial immunological assessments and clinical monitoring being collected. Inclusion criteria comprised willingness to participate and ability to comply with follow-up assessments. Exclusion criteria included acute illness at the time of enrollment, a life expectancy of less than 6 months, ongoing SARS-CoV-2 infection, or contraindications to venous blood sampling. During the study period, the COVID-19 booster vaccine was offered to participants. Specifically, administration occurred in the weeks immediately preceding enrollment or concurrently with baseline sampling (T0), following the chronological sequence outlined in the main COVAC-3 protocol, as described in detail elsewhere- (Palmieri et al., 2026). A substantial proportion of residents did not receive the vaccine booster during the study period. Demographic and clinical data were collected at baseline and included age, sex, comorbidities, vaccination history, and prior SARS-CoV-2 infection. Follow-up data included incident SARS-CoV-2 infections, hospitalizations, mortality, and any new medical conditions. To ensure data quality in this frail population, all baseline and follow-up clinical and demographic data were directly recorded and managed by trained healthcare personnel at each facility through REDCap (Research Electronic Data Capture) electronic data capture tools hosted by the Italian Society of Gerontology and Geriatrics (SIGG) (Harris et al., 2019).

### 2.2 Sample collection and serology

Blood samples were collected at predefined time points: baseline (T0), after 3 months (T1), after 6 months (T2), and after 12 months (T3). Serum was isolated locally, than aliquots were shipped and stored at −80 °C until serological analyses performed at the ISS centralized laboratory. Quantitative IgG antibodies against the SARS-CoV-2 Spike (anti-S) trimeric protein were measured in all participants by the LIAISON® SARS-CoV-2 TrimericS IgG chemiluminescent immunoassay (DiaSorin, Saluggia, VC, Italy); a detailed description can be found elsewhere (Fedele et al., 2022a). The detection of IgG antibodies anti-Nucleocapsid (anti-N) was carried out using the EUROIMMUN Anti-SARS-CoV-2 NCP ELISA (IgG) (EUROIMMUN Medizinische Labordiagnostika AG). This assay is designed for the *in vitro* semi-quantitative detection of IgG antibodies targeting the nucleocapsid protein of SARS-CoV-2 in human serum. The results are calculated as the ratio of the optical density (OD) of the patient sample to the calibrator OD (Lustig et al., 2021). Anti-N seropositivity (cutoff ratio ≥1.0) was utilized as an operational indicator of prior SARS-CoV-2 infection. Results are interpreted as negative for ratios <1, and positive for ratios ≥1. This methodological choice aims to minimize the impact of borderline results on the overall model variance. The threshold of 1.0 was selected as the midpoint of the manufacturer’s indeterminate zone, and was applied to handle borderline results within a binary classification framework for subsequent analyses.

### 2.3 Variables considered and statistical analysis

Variables considered for dimensionality reduction and clustering included demographics, vaccination metrics and longitudinal serological titers. Data were converted to numerical formats, with non-parsable entries treated as missing values. Antibody titers exhibited a highly skewed distribution and were then log-transformed after offsetting zero values to stabilize variance. The relative contribution of each variable to the principal components was evaluated based on its factor loading. Missing data patterns were evaluated prior to dimensionality reduction. To prevent loss of information, missing values in the feature set were imputed using k-Nearest Neighbors (k-NN) imputation (k = 5) prior to Z-score standardization and PCA.

PCA was performed to reduce data dimensionality and identify the main sources of variability across the immunological and clinical variables included in the analysis (Jolliffe and Cadima, 2016). Variables included in the feature matrix for PCA comprised continuous quantitative log-transformed anti-S IgG titers and continuous semi-quantitative anti-N IgG ratios across the longitudinal time points (T0, T1, T2, T3), alongside baseline demographic and clinical covariates (age, sex, comorbidity burden, and vaccination metrics). The adequacy of the correlation structure for PCA was assessed using preliminary diagnostic measures. First, sampling adequacy was evaluated using the Kaiser–Meyer–Olkin (KMO) index, calculated both globally and for individual variables. Individual variables were evaluated using the anti-image correlation matrix; variables with an individual KMO <0.50 (specifically, ‘covid infection 6m’, ‘Number of chronic conditions’, ‘covid infection 12m’, ‘Sex’) were excluded prior from the analysis. In addition, Bartlett’s test of sphericity was performed to assess whether the correlation matrix significantly differed from an identity matrix. A p-value <0.05 confirmed that the data structure possessed adequate internal correlations for dimensionality reduction. The retained variables were standardized using Z-score normalization (μ = 0, σ = 1) to ensure equal weighting in the PCA regardless of their original units. Component retention was guided by the Kaiser criterion (eigenvalue >1), scree plot inspection, and cumulative explained variance. Variable loadings with an absolute value ≥ 0.4 were considered significant for component interpretation and naming. K-means clustering was applied to the retained principal component scores in order to identify groups of residents sharing similar characteristics. Statistical optimization via internal validity indices (Silhouette, Calinski-Harabasz, Davies-Bouldin, Dunn) established the candidate cluster range (k = 2–6), and the final k was selected prioritizing parsimony and interpretability over minor incremental statistical index gains. Because cluster assignment was based on multidimensional patterns that included longitudinal anti-S antibody measurements, the subsequent mixed-effects analysis was strictly intended as descriptive characterization of antibody trajectories across the data-driven phenotypes rather than an independent validation of cluster differences. Longitudinal differences in anti-S antibody trajectories across clusters were evaluated using linear mixed-effects models. The dependent variable was the log-transformed anti-S IgG antibody titer. Fixed effects included cluster assignment, study time modeled continuously across the four study visits (T0–T3), and a cluster-by-time interaction term to test whether longitudinal slope differed between clusters. Study time was coded as 0, 1, 2, and three for T0, T1, T2, and T3, respectively. A subject-specific random intercept was included to account for within-subject correlation arising from repeated measurements. Cluster-specific marginal slope of time were estimated using post-estimation marginal effects, while model-based predicted marginal means were calculated at each study time point to characterize the longitudinal trajectories. Post-baseline vaccination and incident SARS-CoV-2 infection were not incorporated as time-varying covariates in the primary longitudinal model. Although these events were relatively uncommon, their potential influence on antibody trajectories cannot be excluded. The primary LMM was designed to compare longitudinal trajectories across baseline-defined data-derived profiles rather than to estimate the independent effects of subsequent vaccination or infection. Future studies with more detailed time-varying exposure data should explicitly model these events. Average Marginal Effects (AMEs) were estimated to quantify the average rate of antibody decline within each cluster. Differences in slope between clusters (interaction terms) were formally tested using the Wald test. Internal consistency and cluster stability were further validated through a bootstrapped stability analysis (100 iterations) using the Adjusted Rand Index (ARI). Baseline characteristics were compared across the identified clusters. To assess potential attrition bias, baseline demographic, clinical, and immunological characteristics were compared between participants with an available T3 antibody measurement and those lost to follow-up using Wilcoxon rank-sum tests for continuous variables and Chi-squared or Fisher’s exact tests for categorical variables. Continuous variables were summarized as mean ± standard deviation (SD) or median and interquartile range (IQR), as appropriate. Differences in baseline demographic, clinical, and immunological characteristics across clusters were assessed using the Kruskal–Wallis or Mann-Whitney U tests for continuous variables and Chi-square or Fisher’s exact tests for categorical variables, as appropriate. Pairwise comparisons were performed when appropriate. Statistical significance was set at α = 0.05.

Data cleaning, PCA, and K-means clustering were performed using Python (v3.11.13) with standard scientific libraries, including NumPy, pandas, SciPy, and scikit-learn. Longitudinal modeling of antibody decay to analyze the trajectories of antibody decline across the identified clusters, Linear Mixed-Effects Models (LMM), and marginal effects analyses were implemented in Stata (v18).

## 3 Results

A total of 388 residents were enrolled at baseline. Demographic and clinical characteristics of the study population are summarized in Table 1. The mean age was 84.2 years (SD 8.7), with a predominance of females (71%) and a high burden of multimorbidity, with a mean of 4.0 ± 2.0 chronic diseases per resident (range 0–12). Overall, 191 participants (49.2%) received a booster dose during the 2023/2024 vaccination campaign. More than half of participants (67%) showed serological evidence of prior SARS-CoV-2 infection at baseline, defined by the positivity of anti-N antibodies. Considering time-point assessments, 352 participants completed T1, 314 completed T2, and 250 completed T3. During the 12-month follow-up, 83 residents out of 388 (21%) died, and 56 (14%) withdrew consent. Participant retention was evaluated at T3 (n = 250, 64.4%). Baseline characteristics were largely comparable between participants with and without available T3 measurements, with no significant differences observed for age (p = 0.096), comorbidity burden (p = 0.696), baseline anti-S titers (p = 0.709), sex (p = 0.702), or baseline anti-N positivity (p = 0.740). A minor difference was noted in the mean number of prior vaccine doses (4.60 p.m. 1.00 in non-completers vs. 4.35 p.m. 0.91 in completers; p = 0.024).

**TABLE 1:** Baseline study population characteristics.

Demographics and vaccination history	
Variable	Total population (N = 388)​
Age, years (mean ± SD)	84.2 ± 8.7​
Female sex, n (%)	274 (70.6)​
Vaccine doses received (mean ± SD)​	4.4 ± 0.9​
Booster dose in 2023/2024 season, %​	191(49.2%​)
Prior SARS-CoV-2 infection*, n (%)​	260 (67%)​

Baseline immunological status​	
Antibody Marker​	Baseline value (median (IQR))​
IgG anti-S (BAU/mL)​	6330.0 (1755.5 - 15000.0)​
IgG anti-N (Anti-Nucleocapsid)​	2.72 (0.64 - 5.07)​

Baseline clinical condition and chronic conditions ​	
Comorbidity​	Cases (%)​
Arterial Hypertension​	227 (58.5%)​
Cognitive decline/Dementia​	198 (51%)​
Ischemic heart Disease​	136 (35%)​
Arthrosis​	126 (32.4%)​
Depression​	110 (28.3%)​
Diabetes Mellitus​	82 (21.1%)​
Heart Failure​	73 (18.8%)​
COPD​	68 (17.5%)​
Peripheral Artery Disease​	66 (17%)​
Stroke​	66 (17%)​
Chronic Liver Disease​	62 (16%)​
Solid or Hematologic Malignancy​	34 (8.7%)​
Obesity​	25 (6.4%)​
Chronic kidney Disease​	20 (5.1%)​
Malnutrition​	20 (5.1%)​
Inflammatory bowel Disease​	16 (4.1%)​
Immunopathies​	11 (2.8%)​
Mean number of chronic conditions (mean ± SD)​	4.0 (2.0)

*Prior SARS-CoV-2 infection defined by the positivity of anti-N antibodies (T0)

### 3.1 Longitudinal antibody kinetics

At the population level, anti-S antibody concentrations declined progressively over time. Geometric mean titers were highest at baseline (after booster vaccination period) and decreased at T1, T2, and T3 of follow-up (Figure 1). In particular, longitudinal analysis of the study population at baseline (N = 388) showed a significant progressive decline in anti-S levels over the 12-month period (p < 0.001, Figure 1A). When stratified by receipt of the vaccine booster, participants vaccinated during the 2023/2024 campaign exhibited higher baseline anti-S levels, followed by a steeper decline over time. In contrast, individuals who did not receive a booster showed lower baseline titers (Figure 1B). Among vaccinated participants, those who received the booster more than 1 month prior to enrollment had already reached peak antibody levels at baseline, whereas individuals vaccinated within 1 month before enrollment showed a further increase between baseline and the 3-month visit. Considering PCA, the first three principal components (PCs) accounted for 66.4% of the total variance (PC1: 28.8%, PC2: 21.4%, PC3: 16.2%). Bartlett’s test of sphericity confirmed the suitability of the dataset for dimensionality reduction (p < 0.0001) (Supplementary Table S1). Regarding the contribution of each variable to PC1, PC2, and PC3 (Supplementary Figure S1), PC1 was primarily driven by anti-S titers across all time points, was predominantly driven by the overall magnitude and persistence of the vaccine-induced humoral response. PC2 was dominated by IgG-N titers, was descriptively associated with variability associated with natural SARS-CoV-2 infection. PC3 was mainly influenced by vaccination timing, distinguishing individuals vaccinated within 1 month before baseline from those vaccinated earlier or not boosted during the 2023/2024 campaign. K-means clustering applied to PC1–PC3 scores identified three profiles (Supplementary Figure S2). The multi-index validation provided supportive evidence for the 3-cluster solution (Supplementary Figure S3; Supplementary Table S2). At k = 3, the Silhouette score reached 0.296. The Calinski–Harabasz index (164.6) and Davies–Bouldin index (1.19). While the Dunn Index reached its maximum at k = 5 (0.034), the silhouette profiles for k > 3 suggested progressive over-segmentation, with several clusters becoming narrower (Supplementary Table S2). Considering the internal validation indices together with parsimony and interpretability, k = 3 was retained as the clustering solution. Bootstrapped stability analysis yielded a mean ARI = 0.74 (SD = 0.20), providing supportive evidence of cluster reproducibility. Cluster sizes were well balanced (Cluster 0: n = 130; Cluster 1: n = 125; Cluster 2: n = 133), and baseline characteristics differed significantly across clusters (Table 2).

**FIGURE 1:**
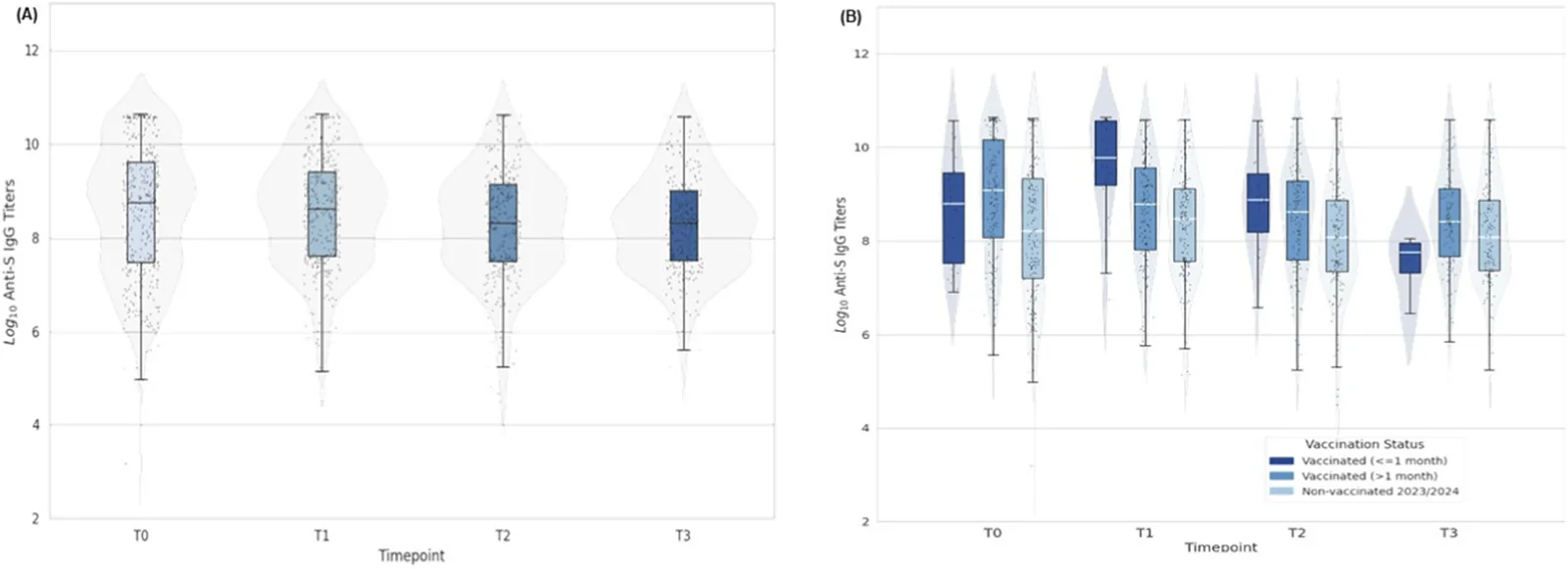
Distribution of anti-Spike IgG levels over time (12-month of follow-up in total). **(A)** Total population kinetics. Log-transformed anti-Spike IgG levels at baseline (T0) and follow-up visits (T1, T2, T3). Individual data points are shown as dots overlaid on boxplots. **(B)** Comparison by vaccination status. Stratified analysis of residents who received the 2023/2024 booster (left) versus non-boosted individuals (right). *Prior SARS-CoV-2 infection defined by the positivity of anti-N antibodies (T0).

**TABLE 2:** Baseline characteristics by cluster.

Variable	Whole sample	Cluster 0 (vaccine-driven)	Cluster 1 (hybrid immunity)	Cluster 2 (high-peak hybrid responders)	*p-value*
N (%)	388	130 (33.5)	125 (32.2)	133 (34.3)	​
Age, years (mean ± SD)​	84.18 ± 8.69	81.90 ± 9.37	85.89 ± 8.23	84.80 ± 7.98	**0.0009**
Female n (%)​	274 (70.5%)	87 (67%)	92 (74%)	94 (71%)	0.4457
Number of chronic conditions median (IQR)	4.0 (3.0–5.0)	4.0 (3.0–5.0)	3.0 (2.0–4.0)	4.0 (3.0–6.0)	**0.0037**
Number vaccine doses (mean ± SD)​	4 ± 0.95	4.35 ± 0.95	3.87 ± 0.86	4.92 ± 0.73	**<0.001**
Vaccinated 2023/24 n (%)	191 (49.2%)	55 (42.3%)	3 (2.4%)	133 (100.0%)	**<0.001**
IgG-S T0 BAU/mL median (IQR)​	6330 (1755.5–15000)	3100 (1520–9140)	4260 (1170–12400)	10700 (5480–28800)	**<0.001**
IgG-N T0 median (IQR)​	2.75 (0.63–5.01)	0.47 (0.24–0.99)	4.4 (2.53–6.28)	3.8 (2.42–6.1)	**<0.001**
Arterial hypertension*	227 (66.8%)	73 (65.8%)	68 (70.1%)	86 (65.2%)	0.7075
Ischemic heart disease*	136 (40.0%)	42 (37.8%)	37 (38.1%)	57 (43.2%)	0.6337
Peripheral artery disease*	66 (19.4%)	20 (18.0%)	16 (16.5%)	30 (22.7%)	0.4509
Heart Failure*	73 (21.5%)	24 (21.6%)	17 (17.5%)	32 (24.2%)	0.4728
Stroke*	66 (19.4%)	17 (15.3%)	18 (18.6%)	31 (23.5%)	0.2677
Diabetes*	82 (24.1%)	30 (27.0%)	26 (26.8%)	26 (19.7%)	0.3158
Depression*	108 (31.8%)	37 (33.3%)	25 (25.8%)	46 (34.8%)	0.3148
Solid or Hematologic Malignancy*	34 (10.0%)	10 (9.0%)	7 (7.2%)	17 (12.9%)	0.3376
Inflammatory bowel Disease*	15 (4.4%)	3 (2.7%)	3 (3.1%)	9 (6.8%)	0.2252
Arthrosis*	126 (37.1%)	43 (38.7%)	30 (30.9%)	53 (40.2%)	0.3265
COPD*	68 (20.0%)	27 (24.3%)	14 (14.4%)	27 (20.5%)	0.2026
Chronic Liver Disease*	62 (18.2%)	14 (12.6%)	20 (20.6%)	28 (21.2%)	0.1731
Chronic kidney Disease*	20 (5.9%)	7 (6.3%)	3 (3.1%)	10 (7.6%)	0.3529
Obesity*	25 (7.4%)	11 (9.9%)	7 (7.2%)	7 (5.3%)	0.3902
Malnutrition*	20 (5.9%)	6 (5.4%)	3 (3.1%)	11 (8.3%)	0.2416
Cognitive decline/Dementia*	198 (58.2%)	59 (53.2%)	47 (48.5%)	92 (69.7%)	**0.0023**
Immunopathies*	12 (3.5%)	6 (5.4%)	1 (1.0%)	5 (3.8%)	0.2286

*P*-values were derived from Kruskal-Wallis test for continuous variables and Chi-square tests for categorical variables.

P-values across baseline characteristics are intended for exploratory profiling and were not adjusted for multiple comparisons.

* Percentages calculated on valid non-missing cases (N = 340).

Bold values indicate statistically significant results (p < 0.05).

### 3.2 Determinants of antibody persistence and waning across profiles

Longitudinal analysis of anti-S antibody trajectories, considering cluster 0 as the reference cluster, revealed heterogeneity the estimated longitudinal trajectories across data-derived groups (Table 3). While the reference cluster showed a modest but significant decline over time (β = −0.031 per follow-up unit, p = 0.042), Cluster 2 × Time interaction was negative and statistically significant (β = −0.057, p = 0.009), although this behavior may be favored by an initial value of the immune response significantly higher than the reference cluster. Average marginal effects showed Cluster 2 had the steepest decline (AME = −0.088, p < 0.001) compared with the reference cluster (Table 4). Figure 2 illustrates the estimated longitudinal anti-S antibody trajectories across the three data-derived groups. Cluster 0 showed a significant decline in IgG-S levels over time. Cluster 1 displayed a flatter trajectory, with no significant change in antibody levels during follow-up, indicating relative stability. In contrast, Cluster 2 showed a significantly steeper observed longitudinal decline than Cluster 0, despite maintaining higher absolute antibody levels throughout the observation period. To evaluate whether the data-driven stratification corresponded to distinct clinical patterns, incident SARS-CoV-2 infections and related clinical events were tracked over the 12-month follow-up period. Overall, the occurrence of documented clinical manifestations of infection was extremely low: only 12 out of 388 residents (3.1%) developed symptomatic SARS-CoV-2 infection during follow-up. Crucially, no COVID-19-related deaths were recorded in the cohort. The 12 incident cases were distributed across the three groups, and no statistically significant association between baseline cluster membership and incident symptomatic infection was detected. Given the very low number of events, this analysis had limited statistical power to detect differences in clinical outcomes across clusters.

**TABLE 3:** Linear random intercept mixed-effects model for longitudinal anti-Spike IgG levels.

Variable	β coefficient	SE	95% CI	*p*-value
Intercept (T0, Cluster 0)	3.54	0.04	3.45–3.62	**<0.001**
Time (per follow-up unit)	−0.031	0.015	−0.061 to −0.001	**0.042**
Cluster 1 (hybrid immunity)	0.09	0.06	−0.03–0.21	0.135
Cluster 2 (high-peak hybrid Responders)	0.48	0.06	0.37–0.60	**<0.001**
Cluster × Time interaction				
Cluster 1 × time	0.019	0.022	−0.024–0.062	0.389
Cluster 2 × time	−0.057	0.022	−0.100 to −0.014	0.009

The outcome variable was log-transformed anti-Spike IgG concentration.

Time was modeled as an continuous variable corresponding to follow-up visits (T0–T3).

A significant negative Cluster 2 (HPHR) × Time interaction indicates a faster antibody decline compared with the reference cluster.

Bold values indicate statistically significant results (p < 0.05).

**TABLE 4:** Average marginal effects of time on anti-Spike IgG by immunological cluster.

Cluster	AME (β)	95% CI	*p-value*
Cluster 0	−0.031	−0.061 to −0.001	0.042
Cluster 1	−0.012	−0.042 to 0.018	0.43
Cluster 2	−0.088	−0.125 to −0.051	**<0.001**

Average marginal effects derived from linear mixed-effects models.

Cluster-specific slopes represent the estimated change in log-transformed anti-S, IgG per follow-up time unit and were obtained using post-estimation marginal effects (margins Cluster, dydx(time)). For Cluster 0, the slope corresponds to the Time coefficient; for Clusters 1 and 2, it corresponds to the sum of the Time coefficient and the respective Cluster × Time interaction coefficient reported in Table 3.

Bold values indicate statistically significant results (p < 0.05).

**FIGURE 2:**
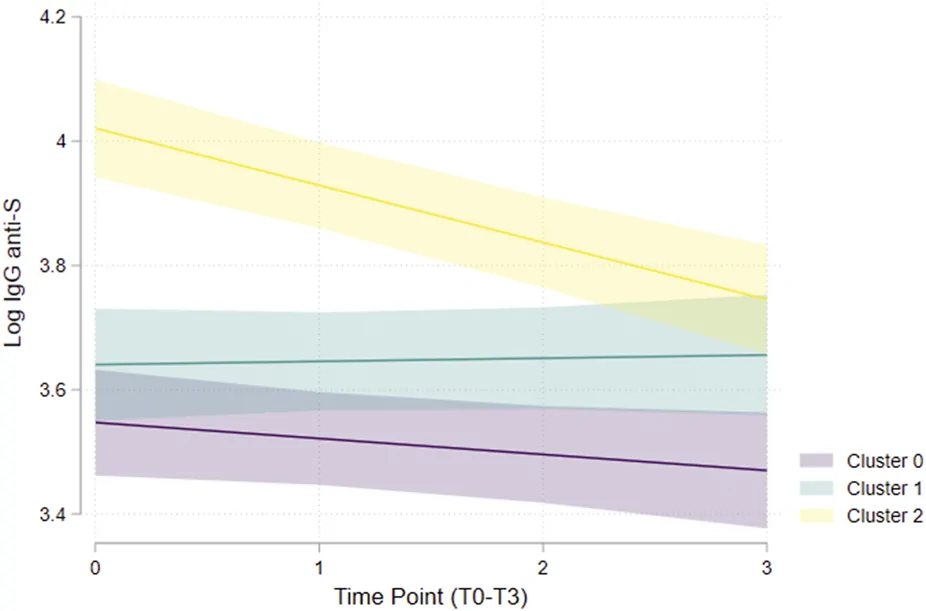
Longitudinal humoral immune trajectories by cluster. Trajectories of log-transformed anti-Spike (anti-S) IgG levels over a 12-month period (T0–T3) using Linear Mixed-Effects Models (LMM). Solid lines represent predicted mean trajectories, and shaded areas indicate 95% confidence intervals.

### 3.3 Clinical and demographic characteristics across clusters

Baseline clinical and demographic characteristics of the three identified clusters are reported in Table 2. Age differed significantly across clusters (p < 0.001), with Cluster 0 including the youngest residents (81.9 ± 9.4 years), whereas Cluster 1 and Cluster 2 included older residents (85.9 ± 8.2 and 84.8 ± 8.0 years, respectively). The number of chronic conditions also differed across clusters (p = 0.0037), with a lower median number of chronic conditions observed in Cluster 1 (median 3, IQR 2–4) compared with Cluster 0 (median 4, IQR 3–5) and Cluster 2 (median 4, IQR 3–6). Vaccination status also differed significantly across clusters (p < 0.001), with vaccination reported in 100% of Cluster 2, compared with 42.3% and 2.4% in Cluster 0 and Cluster 1, respectively. Baseline anti-S IgG titers differed significantly across clusters (p < 0.001), with the highest levels observed in Cluster 2. Similarly, anti-N IgG titers differed significantly across clusters (p < 0.001), with lower levels in Cluster 0 compared with Clusters 1 and 2. Among the clinical characteristics, cognitive decline/dementia was more frequent in Cluster 2 than in Cluster 0 and Cluster 1 (69.7%, 53.2%, and 48.5%, respectively; p = 0.0023). Other baseline comorbidities, including ischemic heart disease, heart failure, stroke, diabetes, depression, malignancies, inflammatory bowel disease, arthrosis, COPD, chronic kidney disease, obesity, malnutrition, and immunopathies, did not significantly differ across clusters (p > 0.05).

## 4 Discussion

In this longitudinal, multicenter study of residents of LTCFs, we characterized humoral immunity to SARS-CoV-2 over a 12-month follow-up period and identified distinct patterns of antibody persistence using an unsupervised, multidimensional approach. Rather than stratifying residents according to predefined clinical or vaccination categories, this data-driven framework allowed data-derived profiles to emerge directly from the multidimensional structure of the dataset. At the population level, anti-S concentrations decreased over time, a trend primarily observed in vaccinated residents, while recent unvaccinated individuals maintained a more stable Anti-S IgG trajectory over time. The integration of serological and vaccination-related variables through PCA and K-means clustering identified three longitudinal antibody response immune profiles, highlighting substantial heterogeneity in humoral immune trajectories among older adults living in LTCFs. PCA reduced the complexity of the multidimensional dataset and identified PC1 reflecting the magnitude of anti-Spike responses, PC2 was descriptively associated with natural exposure to SARS-CoV-2 via anti-N titers, and PC3 discriminating recent booster vaccination from more distant vaccination. K-means clustering applied to these principal component scores further organized residents into three longitudinal data-derived groups, defined primarily by the combined patterns of anti-S responses, anti-N serology and vaccination history. Overall, three longitudinal humoral immune patterns were identified among residents of long-term care facilities that represent descriptive categories: the VD cluster was characterized by moderate anti-Spike titers and limited evidence of natural infection, consistent with a predominantly vaccine-driven immune response. The HI cluster included individuals with clear serological evidence of recent SARS-CoV-2 infection but minimal uptake of the 2023/2024 booster, representing a form of hybrid immunity associated with relatively low but remarkably stable antibody levels over time. The HPHR cluster comprised boosted individuals with hybrid immunity and exhibited the highest anti-Spike antibody concentrations at baseline. Longitudinal modeling using linear mixed-effects was subsequently applied to describe the antibody trajectories across these stratified groups. While a significant overall decline in anti-S levels was observed, the magnitude of waning varied markedly by cluster. Individuals in HPHR group showed the steepest estimated decline in anti-S IgG concentrations over time, despite maintaining higher absolute antibody levels throughout follow-up. The observed pattern is compatible with expected decline following recent antigenic stimulation reported in previous studies of older adults (Saiag et al., 2024; Stirrup et al., 2023; Hofstee et al., 2025; Hirano and Asada, 2024), although the higher baseline antibody levels in this cluster may also contribute to the magnitude of the observed decline. In contrast, the HI group displayed a comparatively flat trajectory, with little evidence of antibody waning across the 12-month period, and the VD group exhibited an intermediate pattern. Average marginal effects quantified these cluster-specific slopes, and further characterized the differences in antibody trajectories observed across the data-driven groups. These findings are consistent with known features of humoral immune responses in older adults, particularly following repeated antigenic stimulation. Importantly, these trajectories emerged without imposing predefined biological assumptions, suggesting that multidimensional unsupervised analyses can reveal exploratory, data-derived profiles within highly heterogeneous older populations. By contrast, individuals with prior infection but without recent boosting maintained lower yet stable antibody levels. Our results suggest that future public health evaluations might benefit from accounting for the underlying heterogeneity of cohort data-derived patterns. Incorporating antibody tracking within multidimensional analytical frameworks, encompassing infection history and longitudinal trajectory patterns rather than relying solely on chronological age or comorbidity count, could refine our understanding of antibody persistence, helping to identify specific sub-populations that may exhibit differences in estimated longitudinal trajectories and benefit most from optimized booster schedules. Several limitations should be acknowledged. First, antibody titers represent only one dimension of immune protection and do not capture functional neutralization capacity or cellular immune responses, which are particularly relevant in older adults. Second, the observational design and real-world vaccination schedules precluded strict standardization of booster timing, potentially contributing to heterogeneity in antibody trajectories. Post-baseline vaccination and incident SARS-CoV-2 infection were not incorporated as time-varying covariates in the primary longitudinal model. During follow-up, 28 participants received an additional vaccine dose and 12 experienced symptomatic infection. These events may have contributed to individual antibody trajectories and therefore cannot be completely excluded as sources of within-cluster heterogeneity. The primary LMM was designed to describe trajectories across baseline-defined cluster rather than to estimate the independent effects of subsequent vaccination or infection. Future studies should incorporate these exposures as time-varying covariates when sufficient longitudinal events are available. Because longitudinal anti-S antibody measurements contributed to cluster construction, the subsequent LMM analysis should therefore be considered a descriptive characterization of trajectories within the data-derived groups rather than an independent validation of the clustering solution. Finally, clinical outcome events were extremely rare. The low number of incident infections limits our statistical power to directly link specific antibody decline slopes to differential risks of clinical breakthrough. In conclusion, this study shows that humoral immunity to SARS-CoV-2 in LTCF residents is characterized by substantial inter-individual heterogeneity that can be effectively organized through longitudinal profiling. Our data show that higher initial antibody titers are followed by a more pronounced absolute decline over time, highlighting the complex interplay between vaccination timing and prior infection history, and longitudinal antibody trajectories in older adults. In our unsupervised analysis, while antibody levels showed a decline following recent booster, the identified data-driven profiles were characterized by combinations of baseline antibody magnitude, prior infection history, and booster status, highlighting the heterogeneity of humoral trajectories in this population. These findings highlight heterogeneity in antibody trajectories in many LTCF residents, with some data-derived groups showing relatively stable antibody concentrations over the follow-up period.

## Data Availability

The raw data supporting the conclusions of this article will be made available by the authors, on request.

## Supplementary material

SUPPLEMENTARY FIGURE S1 Variable loadings on the first three principal components. Heatmap showing the contribution of each variable to PC1, PC2, and PC3. Higher absolute values indicate a stronger influence of the variable on the respective component. PC1 is primarily defined by longitudinal anti-S IgG titers, PC2 by anti-N titers, and PC3 by vaccination timing.

SUPPLEMENTARY FIGURE S2 Principal Component Analysis (PCA) of longitudinal immune responses. Scatter plot showing the distribution of study participants across the first two principal components (PC1 and PC2). Coloured ellipses represent 95% confidence intervals for the three identified clusters.

SUPPLEMENTARY FIGURE S3 Cluster Validation Indices. Multi-index evaluation of k-means clustering performance for k values ranging from 2 to 6, applied to the longitudinal immunological scores (PC1–PC3). Validation plots display the Silhouette Score (top-left), Davies-Bouldin Index (top-right), Calinski-Harabasz Index (bottom-left), and Dunn Index (bottom-right). The red dashed line marks the selected optimal partition at k = 3.

SUPPLEMENTARY TABLE S2 Numerical summary of all validation metrics Silhouette Score, Davies-Bouldin, Calinski-Harabasz, and Dunn Index.
