# Supplementary material for: Longitudinal immune trajectories following SARS-CoV-2 vaccination in long-term care facility residents: an unsupervised analysis approach
Source: Front Aging. 2026 Oct 19;7:1963384. doi: 10.3389/fragi.2026.1963384 (PMC13724463; doi:10.3389/fragi.2026.1963384)
Supplement: Supplementary file 1 [file Table1.docx]

**Supplementary Table S1**. Eigenvalues and variance explained by Principal Components

Analysis.

| Component | Eigenvalue | Variance Explained (%) | Cumulative Variance (%) |
| --- | --- | --- | --- |
| PC1 | 3.4613 | 28.8% | 28.8% |
| PC2 | 2.5766 | 21.4% | 50.2% |
| PC3 | 1.9488 | 16.2% | 66.4% |
| PC4 | 0.9870 | 8.2% | 74.6% |
| PC5 | 0.7696 | 6.4% | 81% |
| PC6 | 0.7374 | 6.1% | 87.1% |
