# Supplementary material for: Longitudinal immune trajectories following SARS-CoV-2 vaccination in long-term care facility residents: an unsupervised analysis approach
Source: Front Aging. 2026 Oct 19;7:1963384. doi: 10.3389/fragi.2026.1963384 (PMC13724463; doi:10.3389/fragi.2026.1963384)
Supplement: Supplementary file 2 [file Table2.docx]

**Supplementary Table S2**. Numerical summary of all validation

metrics Silhouette Score, Davies-Bouldin, Calinski-Harabasz, and Dunn

Index.

| **Number of Clusters (k)** | **Silhouette Score** | **Davies-Bouldin** | **Calinski-Harabasz** | **Dunn Index** |
| --- | --- | --- | --- | --- |
| 2 | 0.270 | 1.48 | 156.8 | 0.026 |
| 3 | **0.296** | **1.19** | **164.6** | **0.021** |
| 4 | 0.301 | 1.13 | 168.1 | 0.031 |
| 5 | 0.315 | 1.01 | 172.7 | 0.034 |
| 6 | 0.329 | 0.94 | 175.6 | 0.019 |
